# The association between manual handling operations and pain in the hands and arms in the context of the 2018 BIBB/BAuA Employment Survey

**DOI:** 10.1186/s12891-021-04495-z

**Published:** 2021-07-30

**Authors:** Charlotte Mueller, Martha Sauter, Julia Barthelme, Falk Liebers

**Affiliations:** 1 Federal Institute for Occupational Safety and Health (BAuA), Noeldnerstr 40/42, 10317 Berlin, Germany; 2 Charité-Universitaetsmedizin Berlin, Berlin, Germany

**Keywords:** Manual handling operations, Musculoskeletal disorders, Upper extremities, Job, Occupational groups

## Abstract

**Background:**

Manual handling operations (MHO) are tasks performed by hand that require repetitive and forceful hand and arm movements. MHO are currently performed in many workplaces in skilled and unskilled jobs in the production and service sectors. MHO are considered as work-related health risk factors. The relationship between MHO and the occurrence of disorders of the upper extremities has been established. MHO can cause diseases such as tenosynovitis or carpal tunnel syndrome. This study aims to assess the current prevalence of MHO in the German workforce and to evaluate the relationship between MHO and the occurrence of hand and arm complaints.

**Methods:**

The analysis was based on the German *2018 BIBB/BAuA Employment Survey*. For this analysis we included subjects aged between 16 and 66 who work at least 35 h per week. The self-reported frequency of MHO (never; rarely; sometimes; often) was considered as the exposure of interest and was stratified by gender and occupation. Prevalence ratios (PR) were used to report the relationship between MHO and self-reported pain in the hands and arms (robust log-linear Poisson regression). Adjustments were made for age, gender, actual weekly working hours, psychosocial workload, and other physical workloads. The regression analyses considered complete cases.

**Results:**

The analyses included 14,299 employees. Frequent MHO were reported by 32.6% of men and 31.1% of women. These workloads were often reported by respondents who work in the agricultural sector (men: 70.1%; women: 79.0%), in unskilled (men: 59.4%; women: 66.9%), and skilled manual occupations (men: 72.7%; women: 66.7%).

A higher frequency of self-reported MHO was associated with a higher prevalence of hand complaints (PR 2.26 CI 2.00–2.55 “often” vs. “never” = ref.) as well as arm pain (PR 1.73 CI 1.55–1.92 for “often” vs. “never” = ref.).

**Conclusion:**

MHO are still frequent in many occupations. The shown association between MHO and pain in the hands and arms demonstrates the importance of MHO in the current German workforce and the necessity to further develop prevention strategies.

**Supplementary Information:**

The online version contains supplementary material available at 10.1186/s12891-021-04495-z.

## Background

The relationship between work-related manual handling operations (MHO) and the occurrence of musculoskeletal disorders in the hands and forearms is well established [1–3]. However, the literature does not provide a unique definition of MHO. Typically, MHO are described as tasks performed by hand, which includes work activities that demand manual skills; highly repetitive sequences of hand and arm movement; and/or operations that require high degrees of hand and arm force [4]. MHO are characterized by a repetitive as well as static strain of the muscles and ligamental structures. The strain these tasks place on the body depends on the intensity of the required effort, the range of motion as well as the duration and frequency of the movements. MHO is a common physical workload among the European and German working population. In 2017, Eurofound conducted the sixth *European Working Conditions Survey* [5], which reported that 33% of women and 30% of men surveyed answered that performing MHO is a major part of every working day. A similar percentage stated that MHO made up 25 to 75% of their working time.

The European Agency for Safety and Health at Work (EU-OSHA) reported that between 61 and 63% of employees were exposed to repetitive hand or arm movements for at least a quarter of their working time across the EU-28 states between 2005 and 2015 [6]. In Germany, data on MHO are collected regularly through the *BIBB/BAuA Employment Surveys*. Conducted every 6 to 7 years, these surveys aim to collect data on the working population to map the current working situation in Germany. For this purpose, data is collected on vocational training and education, working conditions, health conditions, and complaints. Asked about manual handling operations during the *2012 BIBB/BAuA Employment Survey*, 43.5% of men and 40.5% of women responded that they often perform manual work, which includes manual skills, high sequences of movement, and operations that require a high degree of force [7]. On the other hand, 13.8% of men and 18.8% of women responded that they "often" experienced pain in their hands during or after work.

A high degree of repetitive strain to the hand-arm system can cause degenerative structural changes in the muscles and tendons of the arms and hands, and may lead to specific diseases, for example carpal tunnel syndrome (CTS) [1–3, 8, 9]. Disorders such as CTS and tendonitis of the forearms that are caused by work-related, repetitive MHO are recognized as occupational diseases by law in Germany as well as in other countries. In this context, the German statutory accident insurance approved CTS as an occupational disease in 304 out of 1033 cases in 2018 [6, 10].

The etiology of musculoskeletal disorders is considered to be multifactorial [11]. Such other factors besides MHO are related to disorders, complaints, or pain in the hands and arms. There is evidence that other types of physical workload, such as the manual lifting of heavy loads [1–3, 8], and overhead work [1, 9], are risk factors for disorders in the hands and forearms. Furthermore, it has been shown that MHO are also related to physical exposures such as hand-arm vibrations [1, 2, 8], and climatic aspects [12–15]. Individual factors, including age [3, 16], gender [17, 18], being overweight [16, 19, 20], and the patient’s smoking behavior [21], are also described as risk factors for pain in the hands and arms. In some cases, psychosocial factors [8, 22], and the socioeconomic status [23, 24] have also been shown to be related to musculoskeletal disorders of the hands and arms.

The continuous change of current workplaces and the high numbers of employees who perform MHO demand that we provide updated information on the distribution, the impact and the consequences of MHO to the health of employees in today’s workplaces. Therefore, this study aimed to describe the self-reported prevalence of exposure to manual handling operations in the current German workforce based on the *2018 BIBB/BAuA Employment Survey* and to investigate the relationship between the intensity of manual handling operations and the prevalence of pain in the hands and arms to estimate the current percentage of pain prevalence attributable to MHO.

## Methods

### Study design and study population

The study was part of the F2456 project of the German Federal Institute for Occupational Safety and Health (BAuA). The study is a secondary analysis using the data of the *2018 BIBB/BAuA Employment Survey* with an emphasis on exposure to physical workloads and its association to musculoskeletal disorders. The *2018 BIBB/BAuA Employment Survey* was based on telephone interviews and used a cross-sectional study design. German-speaking people aged 15 and older who work more than 10 h per week were included in the survey [25]. BAuA entrusted social research company Kantar Public with the conduct of standardized telephone interviews between October 2017 and April 2018. The sampling strategy of the survey used a dual frame sampling approach, which included landline and cell phone numbers to reach a wide range of participants. To increase the availability of the employees, the interviews were mainly conducted in the afternoons, evenings, and on weekends. The survey covered several confounders such as age, gender, and key aspects of social-economic status. Other epidemiologically relevant facts, including smoking behavior, body weight, and height, were not part of the questionnaire and could therefore not be used. Overall, 20,012 employees were interviewed and included in the *2018 BIBB/BAuA Employment Survey*.

For this paper, the study population was reduced to persons of working age aged < 67 years who work in fulltime jobs (normal working hours of 35 h per week or more). No minors below the age of 16 years were included.

### Response variables: hand and arm pain

Employees were asked about the occurrence of a range of pains and complaints related to or experienced after work, in different regions of the musculoskeletal system that occurred within the past 12 months, with possible answers being “yes” and “no”. The answers relating to hand and arm pain were used as separate outcomes of interest.

### Explanatory variables: manual handling operations and covariates

Furthermore, participants were asked about different aspects of their current working conditions. The frequency of manual handling operations was assessed via the following question: “Do you perform work with your hands that includes intensive manual work, fast sequences of movement, or requires high levels of force?” (Author’s translation). Participants could give one of four categories as a response, namely “never”, “rarely”, “sometimes” or “often”. If respondents refused to answer, this was treated as a missing answer and was excluded from the analysis.

The question regarding the manual lifting of heavy loads was different for men and women with respect to the absolute weight of the load lifted (men: 20 kg or more; women: 10 kg or more). Other physical exposures at the workplace were examined in a similar manner to MHO; only “overhead work” was indirectly derived and used in the regression models as a binary dummy variable. This variable was generated based on the answers related to the frequency of work performed in awkward body postures. Similar to the manual lifting of loads, employees were asked to state whether their work required them to “never”, “rarely”, “sometimes” or “often” work in awkward postures such as bending, working overhead, or kneeling or squatting. If interviewees answered “often”, they were subsequently asked in which of these awkward postures they often worked. A binary dummy variable was then developed from the combination of these answers (often working overhead: “yes” or “no”). Important confounders such as age (years) and gender (men; women) were surveyed at the beginning of the interview. Actual working time per week (hours) included overtime, extra work, and on-call service. Working hours in other part-time jobs were not considered.

To assess the psychosocial workload, a score was calculated based on the Kroll index [26]. This index represents the psychosocial workload of employees and considers different psychosocial aspects, which were covered in the *2018 BIBB/BAuA Employment Survey*. Subcategories of the psychosocial workload index include psychological stress, social burdens, and temporal involvement. These sub-indices were calculated by totaling the points of the corresponding single items according to the answers given. The sub-indices of the three subcategories were totaled and then divided by the maximum number of achievable points of all validly answered individual items. The average of the three scores of the subcategories were interpreted as the psychosocial workload index; this index ranges from 0 to 100 points.

The occupations of the participants in the *2018 BIBB/BAuA Employment Survey* available in the dataset had been coded in accordance with a German classification of occupations. The coded job titles were grouped and assigned to twelve main occupational groups using the job classification published by BLOSSFELD in 1985 [27] (Production: agricultural occupations; unskilled manual occupations; skilled manual occupations; technicians; engineers; Service: unskilled services; skilled services; semiprofessions; professions; Administration: unskilled commercial and administrational occupations; skilled commercial and administrational occupations; managers).

### Data privacy and ethics

In March 2017, the BAuA ethics committee approved the design and methods of the *2018 BIBB/BAuA Employment Survey*. The dataset was provided in an anonymized manner. All personally identifiable information was removed from the dataset.

### Statistical methods

The study used descriptive statistics and inference statistical approaches. Absolute and relative frequencies have been presented for the categorical variables, while the arithmetic mean and standard deviation (SD) have been provided for numeric variables. Generally, the descriptive statistics for all items used are shown stratified by the four categories of self-reported frequency of MHO (“never”, “rarely”, “sometimes”, and “often”). We reported the prevalence of the exposure variable MHO stratified by gender and for all BLOSSFELD occupational groups.

Loglinear Poisson regression analyses with robust variance estimation (generalized linear models, SPSS 25®, GENLIN) were conducted to assess the association between MHO and hand and arm complaints. The effect estimates were interpreted as prevalence ratios (PR). The marginal means of the outcome prevalence are provided for the exposure categories “never”, “rarely”, “sometimes” and “often”. The calculation of these means was based on post-estimations using the results of the fully adjusted regression Model #5. As a result, we have provided the adjusted prevalence stratified by the self-reported frequency of MHO, assuming that influencing categorical variables were evenly distributed [28]. Metric variables were centered (age = 45 years, weekly working hours = 40 h, psychosocial workload = 38.9 index points) [29]. The difference of the adjusted prevalence between the exposure categories can be interpreted as the absolute proportion of the prevalence which is attributable to the exposure.

A literature search [1–3, 8, 9, 12–24] was conducted to identify essential factors that may impact the outcome. The a-priori defined list of covariates were included in an underlying causal diagram and were considered in the regression models [30]. In addition to MHO, the following cofactors that were available in the dataset were included: age; gender; weekly working time; overhead work; manual lifting of heavy loads; climatic factors (“cold, heat, wet humidity, draught”); and psychosocial workload. The covariates were added to the regression models block by block:

- Model #1: MHO only;
- Model #2: #1 with age, gender;
- Model #3: #2 and weekly working time;
- Model #4: #3 and overhead work, manual lifting of heavy loads, climatic factors;
- Model #5: #4 and psychosocial workload (fully adjusted model).
- Model #6: #5 and interaction terms for MHO x Gender

This study will focus on two of these five models: the unadjusted Model #1, which only includes MHO as an exposure of interest, and the fully adjusted Model #5, which includes all listed variables. Based on the fully adjusted Model #5 we analyzed for both outcomes in Model #6 the interaction between manual handling operations (4 response categories) and gender (2 categories) using three interaction terms (“rarely MHO” x women; “sometimes MHO” x women; “often MHO” x women).

To assure each regression model has the same number of participants, only complete cases were considered. We selected datasets which were complete with regard to the exposure of interest, the two selected outcomes of interest (arm and hand pain) and all covariates. As a result, cases with missing variables have been excluded [31].

The post-estimation of the prevalence of hand and arm complaints for all categories of exposure to MHO was based on the results of the regression models and allowed for adjusted estimates of the outcome prevalence per exposure category with 95% confidence intervals (95% CI). Syntax-based statistical calculations were conducted using IBM SPSS Statistics 25.

## Results

### Study population

The *2018 BIBB/BAuA Employment Survey* included 20,012 subjects. After applying the inclusion criteria, the sample size was reduced to 14,414 subjects. Of these, 14,375 employees responded to the question on hand pain, and 14,372 to the question on arm pain. Considering these two outcomes as well as all other variables included in the regression analyses, 14,299 cases with complete datasets were available and used for the analyses (Additional Table 1). The analyzed sample of 14,299 employees included 8,809 men (61.6%) men and 5,490 women (38.4%). The mean age of the subjects was 46.7 (SD 11.1); respondents reported a mean actual working time per week of 43.8 (SD 7.6) hours. The mean of the psychosocial workload index based on Kroll 2011 [26] was 38.9 (SD 11.8) index points (Table 1).

**Table 1:** Description of the study population stratified by the self-reported frequency of manual handling operations

	**Self-reported frequency of manual handling operations**				
	**Never**	**Rarely**	**Sometimes**	**Often**	**Total**
**Manual handling operations**					
* All participants (n (row%)	6119 (42.8%)	1677 (11.7%)	1922 (13.4%)	4581 (32.0%)	14,299 (100.0%)
* Men (n (row%))	3476 (39.5%)	1183 (13.4%)	1276 (14.5%)	2874 (32.6%)	8809 (100.0%)
* Women (n (row%))	2643 (48.1%)	494 (9.0%)	646 (11.8%)	1707 (31.1%)	5490 (100.0%)
**Age (years, mean (SD))**	47.6 (10.7)	46.4 (11.2)	46.0 (11.3)	45.8 (11.5)	46.7 (11.1)
**Women (%)**	43.2%	29.5%	33.6%	37.3%	38.4%
**Weekly working hours (mean (SD))**	43.5 (6.8)	44.2 (7.4)	43.8 (8.1)	44.0 (8.4)	43.8 (7.6)
**Psychosocial workload index (index points, mean (SD))**	36.4 (10.5)	39.0 (11.4)	40.3 (12.3)	41.6 (12.7)	38.9 (11.8)
**Prevalence of hand pain (n (%))**					
Men	175 (5.0%)	95 (8.0%)	141 (11.1%)	641 (22.3%)	1052 (11.9%)
Women	300 (11.4%)	62 (12.6%)	98 (15.2%)	516 (30.2%)	976 (17.8%)
All	475 (7.8%)	157 (9.4%)	239 (12.4%)	1157 (25.3%)	2028 (14.2%)
**Prevalence of arm pain (n (%))**					
Men	261 (7.5%)	122 (10.3%)	194 (15.2%)	762 (26.5%)	1339 (15.2%)
Women	386 (14.6%)	75 (15.2%)	111 (17.2%)	581 (34.0%)	1153 (21.0%)
All	647 (10.6%)	197 (11.7%)	305 (15.9%)	1343 (29.3%)	2492 (17.4%)
**Manual lifting of heavy loads (n (col%))**					
Never	4704 (76.9%)	547 (32.6%)	502 (26.1%)	1000 (21.8%)	6753 (47.2%)
Rarely	994 (16.2%)	825 (49.2%)	562 (29.2%)	871 (19.0%)	3252 (22.7%)
Sometimes	269 (4.4%)	165 (9.8%)	539 (28.0%)	828 (18.1%)	1801 (12.6%)
Often	152 (2.5%)	140 (8.3%)	319 (16.6%)	1882 (41.1%)	2493 (17.4%)
**Overhead operations (n (%))**					
Yes	15 (0.2%)	27 (1.6%)	79 (4.1%)	592 (12.9%)	713 (5.0%)
**Cold; heat; wet humidity; draught (n (col%))**					
Never	4899 (80.1%)	702 (41.9%)	721 (37.5%)	1559 (34.0%)	7881 (55.1%)
Rarely	530 (8.7%)	485 (28.9%)	303 (15.8%)	578 (12.6%)	1896 (13.3%)
Sometimes	455 (7.4%)	265 (15.8%)	583 (30.3%)	878 (19.2%)	2181 (15.3%)
Often	235 (3.8%)	225 (13.4%)	315 (16.4%)	1566 (34.2%)	2341 (16.4%)

*row%* Row percentage, *col%* Column percentage, *%* Percentage, *n* Absolute number, *SD* Standard deviation

### Unadjusted prevalence of manual handling operations

A share of 32.6% of men and 31.1% of women indicated that they often perform MHO. Employees who reported that they often perform MHO also often lifted heavy loads (41.1%), often worked overhead (12.9%) or reported that they often work under cold climatic conditions, in heat, wet humidity, or draught (34.2%).

Men and women employed in occupations in the agricultural sector (e.g., farmers and forest workers), in unskilled manual occupations (e.g., construction helpers and road builders) or in skilled manual occupations (e.g., electricians and carpenters) reported that they frequently executed MHO. Women in semiprofessions (e.g., nurses) another large and important occupational group are often exposed to MHO. The distribution of the prevalence of manual handling operations in occupational sectors stratified by gender is provided in Table 2.

**Table 2:** Prevalence of manual handling operations stratified by the BLOSSFELD occupational groups and gender

**BLOSSFELD occupational group**	**Self-reported frequency of manual handling operations**				
	Never n (row %)	Rarely n (row %)	Sometimes n (row %)	Often n (row %)	Total n (row %)
**Men (*n* = 8770)**					
Agricultural occupations	10 (5.3%)	13 (7.0%)	33 (17.6%)	131 (70.1%)	187 (100.0%)
Unskilled manual occupations	79 (11.7%)	72 (10.7%)	122 (18.1%)	400 (59.4%)	673 (100.0%)
Skilled manual occupations	50 (4.1%)	81 (6.7%)	198 (16.4%)	878 (72.7%)	1207 (100.0%)
Technicians	175 (28.2%)	106 (17.1%)	125 (20.1%)	215 (34.6%)	621 (100.0%)
Engineers	428 (55.7%)	147 (19.1%)	111 (14.5%)	82 (10.7%)	768 (100.0%)
Unskilled services	166 (19.2%)	144 (16.6%)	174 (20.1%)	382 (44.1%)	866 (100.0%)
Skilled services	163 (29.8%)	85 (15.5%)	105 (19.2%)	194 (35.5%)	547 (100.0%)
Semiprofessions	234 (40.8%)	101 (17.6%)	102 (17.8%)	137 (23.9%)	574 (100.0%)
Professions	291 (54.6%)	67 (12.6%)	69 (12.9%)	106 (19.9%)	533 (100.0%)
Unskilled commercial and administrational occupations	95 (42.8%)	34 (15.3%)	37 (16.7%)	56 (25.2%)	222 (100.0%)
Skilled commercial and administrational occupations	1054 (66.9%)	197 (12.5%)	126 (8.0%)	198 (12.6%)	1575 (100.0%)
Managers	712 (71.4%)	130 (13.0%)	67 (6.7%)	88 (8.8%)	997 (100.0%)
**All men**	3457 (39.4%)	1177 (13.4%)	1269 (14.5%)	2867 (32.7%)	8770 (100.0%)
**Women (*n* = 5473)**					
Agricultural occupations	5 (8.1%)	1 (1.6%)	7 (11.3%)	49 (79.0%)	62 (100.0%)
Unskilled manual occupations	19 (12.3%)	12 (7.8%)	20 (13.0%)	103 (66.9%)	154 (100.0%)
Skilled manual occupations	14 (8.6%)	8 (4.9%)	32 (19.8%)	108 (66.7%)	162 (100.0%)
Technicians	65 (34.2%)	20 (10.5%)	23 (12.1%)	82 (43.2%)	190 (100.0%)
Engineers	82 (59.9%)	22 (16.1%)	16 (11.7%)	17 (12.4%)	137 (100.0%)
Unskilled services	55 (24.6%)	21 (9.4%)	30 (13.4%)	118 (52.7%)	224 (100.0%)
Skilled services	121 (26.1%)	55 (11.9%)	66 (14.2%)	222 (47.8%)	464 (100.0%)
Semiprofessions	542 (38.2%)	143 (10.1%)	248 (17.5%)	486 (34.2%)	1419 (100.0%)
Professions	316 (59.8%)	57 (10.8%)	54 (10.2%)	101 (19.1%)	528 (100.0%)
Unskilled commercial and administrational occupations	116 (42.8%)	19 (7.0%)	30 (11.1%)	106 (39.1%)	271 (100.0%)
Skilled commercial and administrational occupations	871 (65.4%)	106 (8.0%)	99 (7.4%)	256 (19.2%)	1332 (100.0%)
Managers	427 (80.6%)	30 (5.7%)	20 (3.8%)	53 (10.0%)	530 (100.0%)
**All women**	2633 (48.1%)	494 (9.0%)	645 (11.8%)	1701 (31.1%)	5473 (100.0%)
**All men and women**	**6090 (42.8%)**	**1671 (11.7%)**	**1914 (13.4%)**	**4568 (32.1%)**	**14,243 (100.0%)**

*row%* Row percentage

### Crude prevalence of hand and arm complaints

The crude prevalence of self-reported hand pain within the past 12 months is 14.2%. Hand pain is generally more common in women than in men (17.8 vs. 11.9%).

For both genders the prevalence increased in relation to the self-reported frequency of manual handling operations. Nearly 11.4% of women who reported that they “never” perform MHO experienced hand pain within the previous 12 months. In women, the prevalence increased over the following categories of frequency of MHO, with 12.6% in the category “rarely” 15.2% in the category “sometimes” and 30.2% in the category “often”. Men demonstrated a similar trend. The prevalence of a self-reported, 12-month prevalence of hand pain increased from 5.0% for men who reported that they “never” perform MHO, to 8.0 and 11.1% for men who were “rarely” and “sometimes” exposed, and to 22.3% for men in the highest exposure category (Table 1).

Regarding arm pain, the second outcome, 17.4% of subjects reported that they had suffered such complaints within the past 12 months. Generally, women more frequently expressed the occurrence of arm pain than men (21.0 vs. 15.2%). There was a strong increase in the prevalence of arm pain in relation to the self-reported frequency of manual handling operations from 10.6% in subjects who were never exposed to manual handling operations to 29.9% in subjects who were often exposed. This increase of prevalence related to the exposure to manual handling operations was found for men as well as for women (Table 1).

### Association between manual handling operations and hand complaints

**Fig. 1:**
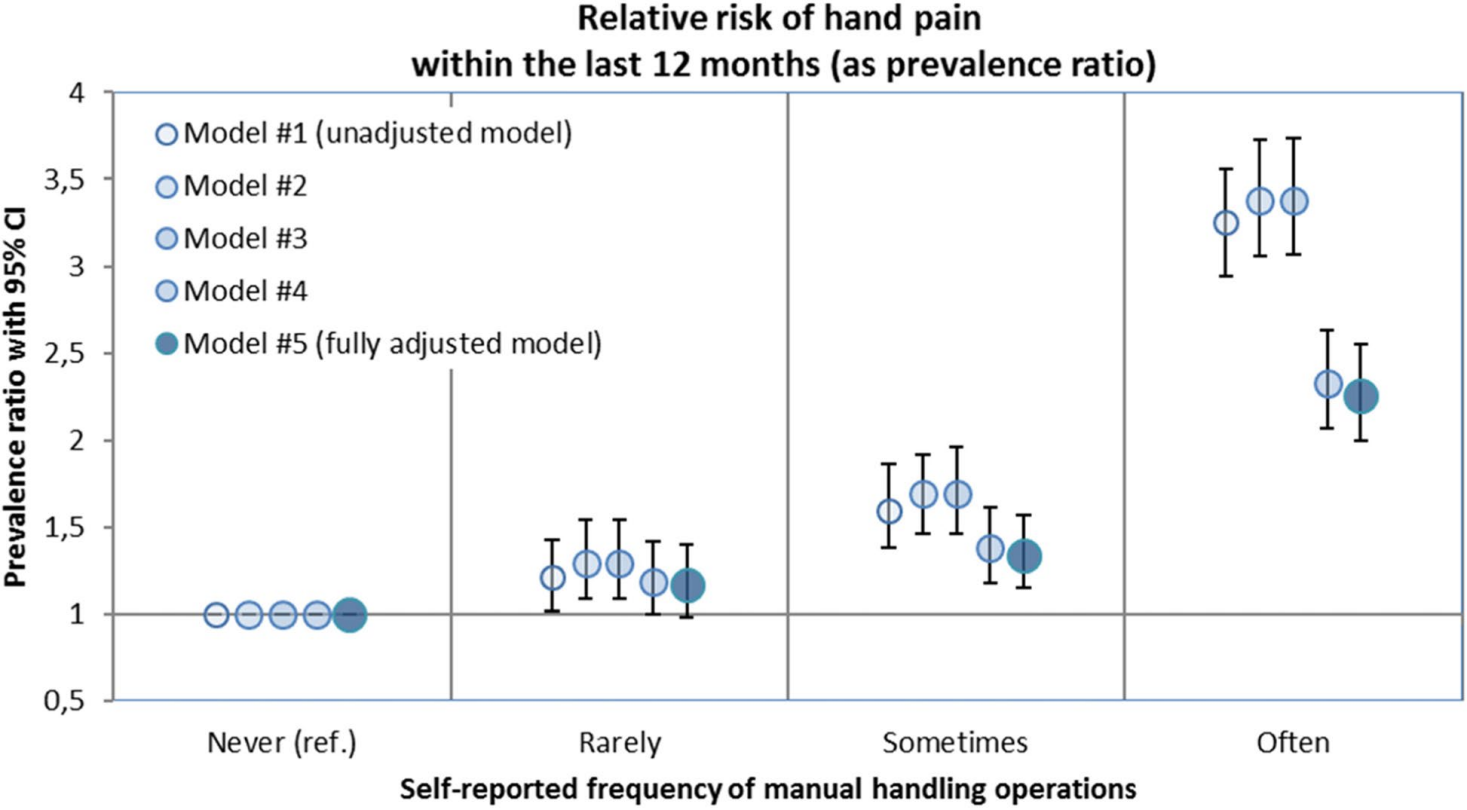
Relative risk of self-reported of hand pain or complaints stratified by the self-reported frequency of manual handling operations. Prevalence ratios presented for the unadjusted Model #1 and the adjusted regression Models #2 to #5 (reference category = “Never performs manual handling operations”)

There was a strong association between MHO and hand pain in the unadjusted as well as in the adjusted models. All models showed a strong increase of the PR related to the increase of the self-reported frequency of MHO. In comparison to the adjusted models, the estimated association tended to be higher in the unadjusted model. According to the latter, subjects in the category “rarely performs MHO” had a 1.21-fold (CI 1.02–1.43) increased risk of suffering hand pain compared to subjects in the reference group who reported that they “never perform MHO”. Based on the unadjusted model, the risk of hand pain increased 1.60-fold (CI 1.38–1.86) to 3.25-fold (CI 2.45–3.60), respectively, for employees who stated that they “sometimes” or “often” perform MHO. After adjustment for confounders (age; gender; weekly working time; manual lifting of heavy loads; overhead work; and “cold, heat, wet humidity, draught”), the association between MHO and pain in the hands remained high. Employees who reported that they “rarely perform MHO” had a higher probability of self-reported pain in the hands within the last 12 months. The prevalence of hand pain in the group of employees who “sometimes” perform MHO was 1.34 (CI 1.15–1.57) times higher compared to subjects who “never” perform MHO. The prevalence ratio rose to 2.26 (CI 2.00–2.55) for subjects who reported that they “often” performed MHO (Table 3, Fig. 1). Other relevant physical workloads and exposures, such as working in extreme climatic conditions, were considered in the fully adjusted model. The relative risk for hand pain was associated with a high frequency of self-reported manual lifting of heavy loads (often: PR 1.34 CI 1.18–1.52), overhead work (PR 1.39 CI 1.23–1.56), and often working under difficult climatic factors (PR 1.50 CI 1.34–1.67) (Table 3). Compared to men, women showed a higher probability of self-reported hand pain within the past 12 months (PR 1.59 CI 1.47–1.72). The probability of reported hand pain increased by a factor of 1.10 (CI 1.07–1.15) per 10 years of age.

**Table 3:** Relative risk of hand pain as a prevalence ratio (PR) with a 95% confidence interval (95% CI) in unadjusted Model #1 and fully adjusted Model #5

**Exposure of interest and covariates**	**Categories / Units**	**Unadjusted PR (95% CI) for hand pain (Model #1)**	**Adjusted PR (95% CI) for hand pain (Model #5)**
**Manual handling operations**	Often	3.254 (2.945–3.595)	2.260 (2.002–2.551)
	Sometimes	1.602 (1.383–1.855)	1.343 (1.149–1.571)
	Rarely	1.206 (1.015–1.433)	1.172 (0.981–1.400)
	Never (ref.)	1 (ref.)	1 (ref.)
**Manual lifting of heavy loads**	Often		1.341 (1.183–1.520)
	Sometimes		1.149 (1.003–1.317)
	Rarely		0.959 (0.844–1.089)
	Never (ref.)		1 (ref.)
**Overhead work**	Often		1.386 (1.229–1.563)
	Rarely (ref.)		1 (ref.)
**Cold; heat; wet humidity; draught**	Often		1.496 (1.340–1.669)
	Sometimes		1.191 (1.056–1.343)
	Rarely		0.985 (0.856–1.133)
	Never (ref.)		1 (ref.)
**Age**	Per year		1.010 (1.007–1.014)
**Gender**	Women / men (ref.)		1.587 (1.464–1.721)
**Actual weekly working hours**	Per hour		0.988 (0.982–0.994)
**Psychosocial workload index (Kroll 2011) **[26]	Per index-point		1.012 (1.009–1.016)

The following confounder variables were included in the fully adjusted model: gender; manual lifting of heavy loads; overhead work; climatic factors; age; actual weekly working hours; and an index for psychosocial workload based on Kroll (2011) [26]. The regression models include *N* = 14,299 subjects (complete case analysis)

*ref.* Reference group, *PR* Prevalence ratio, *95% CI* 95% confidence interval

The adjusted prevalence was estimated based on the results of the fully adjusted Model #5. Employees who “often” performed MHO had the highest prevalence of hand pain (25.9% CI 24.0–28.0%). In employees who “sometimes”, “rarely” or “never” performed MHO, the prevalence of pain in the hands was 15.5% (CI 13.5–17.6%), 13.4% (CI 11.4–15.8%) and 13.4% (CI 11.4–15.8%), respectively. Therefore, if we compare subjects who “never performed MHO” to subjects who “often performed MHO”, nearly 12.5% of the total percentage of hand pain prevalence is attributable to the MHO workload.

In Model #6 we added three interaction terms to Model #5 to get estimates for the interaction between gender and the three higher exposure categories of manual handling operations. Comparable to Model #5 without interaction terms, the general estimates in Model #6 were increased for the higher categories of manual handling operations (“rarely MHO”: PR 1.44 CI 1.13–1.84; “sometimes MHO”: PR 1.75 CI 1.40–2.18; “often MHO”: PR 2.91 CI 2.44–3.48) as well as for women (PR 2.19 CI 1.83–2.62). The estimates of the interaction terms were lower than 1 (“rarely MHO” and “woman”: PR 0.71 (CI 0.50–1.00); “sometimes MHO” and “woman”: PR 0.63 (CI 0.47–0.85); and “often MHO” and “woman”: PR 0.65 (CI 0.53–0.80)). As result this pointed to a more additive than multiplicative type of interaction between gender and manual handling operations.

### Association between manual handling operations and arm complaints

**Fig. 2:**
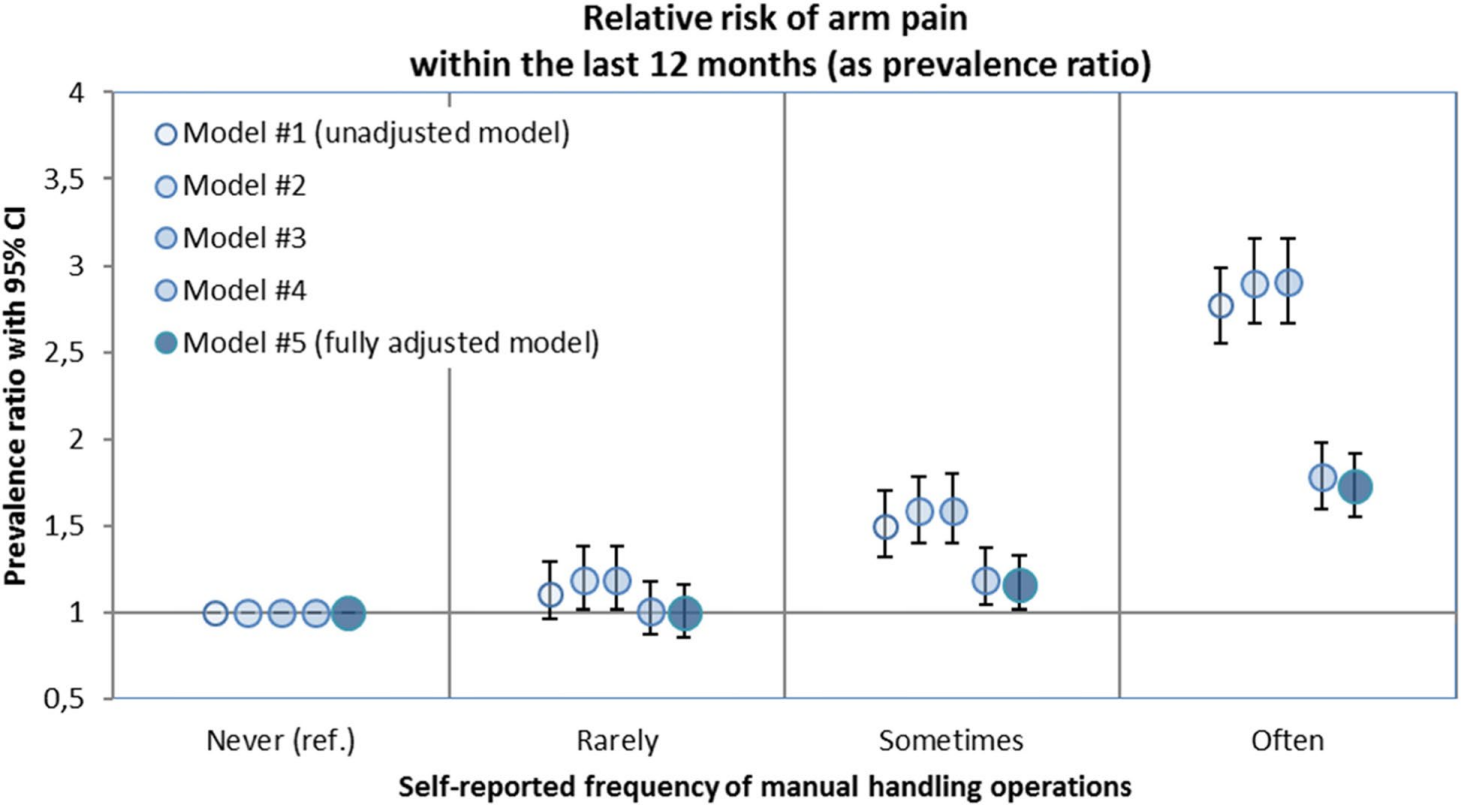
Relative risk of self-reported of arm pain or complaints stratified by the self-reported frequency of manual handling operations Prevalence ratios presented for the unadjusted Model #1 and the adjusted regression Models #2 to #5 (reference category = “Never performs manual handling operations”)

Similar to the results for hand pain as an outcome, we were able to prove a strong association between the self-reported frequency of manual handling operations and the prevalence of arm pain in the unadjusted as well as in the adjusted models. Regarding the unadjusted model the prevalence of arm pain is not significantly increased (PR 1.11 CI 0.96–1.29) in subjects who reported that they are rarely exposed to MHO; 1.50-fold (CI 1.32–1.70) in those who were sometimes exposed; and 2.77-fold (CI 2.54–3.02) in subjects who were often exposed, compared to subjects who were never exposed. The association was less characteristic if confounder variables were included in the regression model. Considering the fully adjusted Model #5 the prevalence ratio was 1.73 (CI 1.55–1.92) in the highest exposure category “often” and 1.16 (CI 1.02–1.33) in subjects who reported that they are sometimes exposed to manual handling operations (Table 4, Fig. 2). For other work related exposures (manual lifting of heavy loads; overhead work; climatic exposures; psychosocial workload; actual weekly working hours) and individual factors (age; gender) we find nearly the same associations to the self-reported frequency of manual handling operations as for hand pain (Table 4). The interaction between gender and self-reported exposure to manual handling operations showed a similar tendency for arm complaints as described for hand pain.

**Table 4:** Relative risk of arm pain or complaints as prevalence ratio (PR) with a 95% confidence interval (95% CI) in the unadjusted and fully adjusted Model #5

**Exposure of interest and covariates**	**Categories / Unit**	**Unadjusted PR (95% CI) for arm pain (Model #1)**	**Adjusted PR (95% CI) for arm pain (Model #5)**
**Manual handling operations**	Often	2.773 (2.545–3.020)	1.728 (1.552–1.923)
	Sometimes	1.501 (1.323–1.703)	1.162 (1.015–1.330)
	Rarely	1.111 (0.956–1.291)	0.996 (0.855–1.159)
	Never (ref.)	1 (ref.)	1 (ref.)
**Manual lifting of heavy loads**	Often		1.590 (1.420–1.781)
	Sometimes		1.205 (1.063–1.366)
	Rarely		1.063 (0.951–1.190)
	Never (ref.)		1 (ref.)
**Overhead work**	Often		1.296 (1.166–1.440)
	Rarely (ref.)		1 (ref.)
**Cold; heat; wet humidity; draught**	Often		1.657 (1.500–1.830)
	Sometimes		1.297 (1.166–1.442)
	Rarely		1.048 (0.925–1.188)
	Never (ref.)		1 (ref.)
**Age**	Per year		1.020 (1.016–1.023)
**Gender**	Women / men (ref.)		1.458 (1.357–1.567)
**Actual weekly working hours**	Per hour		0.987 (0.981–0.992)
**Psychosocial workload index (Kroll 2011) **[26]	Per index-point		1.011 (1.008–1.014)

The following confounder variables were included in the fully adjusted model: gender; manual lifting of heavy loads; overhead work; climate factors; age; actual weekly working hours; and an index for psychosocial workload based on Kroll (2011) [26]. The regression models include *N* = 14,299 subjects (complete case analysis)

*ref.* Reference group, *PR* Prevalence ratio, *95% CI* 95% confidence interval

The adjusted prevalence of arm pain in subjects who never, rarely, sometimes, or often performed MHO was estimated based on regression Model #5 with 16.1% (CI 14.6–17.9%), 16.1% (CI 14.0–18.5%); 18.7% (CI 16.7–21.0%); and 27.9% (CI 26.0–29.9%), respectively. The percentage of arm pain prevalence attributable to the MHO workload is nearly 11.8% when comparing subjects who “never performed MHO” to subjects who “often performed MHO”.

## Discussion

The study aimed to assess the exposure prevalence of manual handling operations (MHO) and the association between MHO and self-reported pain in the hands and arms using the data of the *2018 BIBB/BAuA Employment Survey*. The results show that the prevalence of pain in the hands and arms within the past 12 months strongly increased in relation to the self-reported frequency of manual handling operations (MHO) in men and women. However, pain in the hands and arms is generally more common in women than in men. The results demonstrate that MHO is still a significant risk factor in the German working population. One third of all employed women and men reported that they “often” performed MHO. These highly exposed employees also reported that they are often exposed to other related physical or environmental risk factors such as manual lifting of heavy loads, overhead work or difficult climates. Most employees who worked in the agricultural sector (such as farmers and forest workers) and in unskilled manual occupations (such as construction helpers and road builders) or skilled manual occupations (such as electricians and carpenters) “often” performed manual handling operations.

We were able to show a strong association between the self-reported frequency of MHO and hand and arm pain in the unadjusted and as well as in the adjusted models. After adjusting for relevant confounders, the prevalence ratio for the categories “sometimes” and “often” remains high. The relationship between the self-reported frequency of MHO and hand and arm pain is generally more pronounced for “hand pain” than for the less specific outcome of “arm pain”. According to the results of the regression models, the adjusted prevalence of hand and arm pain increases with the self-reported frequency of manual handling operations.

It must be considered that the implemented analysis is based on a cross-sectional-study; it is therefore not possible to provide causal statements.

One advantage of the study is the large study population of 20,012 employees. 47.4% of reachable employees (*n* = 42,188) participated. A comparison of responders to non-responders is complicated, as basic information (age; education) is only available for 5.1% of them.

Although the percentage of men and women is equally distributed within the German population [32], men were more prevalent than women in our study population. This may be explained by the inclusion criteria applied to this analysis: employees who work less than 35 h per week had been excluded. As a result, many women were not considered in the analysis. But, the restriction regarding working time was introduced to minimize the effect of unmeasured confounding by leisure time activities, gardening or sport activities, which were not assessed in the 2018 *BIBB/BAuA Employment Survey*. Regardless of this restriction adjustment of the regression models taking into account working hours was still necessary due to the remaining variance of weekly working time in the resulting study sample (mean (SD): 43.8 h (7.6 h); median: 40 h; IQR: 12 h).

When interpreting the results, we must consider a healthy worker effect [33]. This effect can impact all categories of MHO. It should be taken into account that employees who remain in occupations with high workloads of MHO adapt to their working conditions, whereas employees with health conditions are more likely to leave the workplace or switch to less exposed fields of work. Therefore, the real effect estimates of MHO with regards to the prevalence of hand pain may be higher.

Another consideration is that the *2018 BIBB/BAuA Employment Survey* covered a wide range of work-related aspects, with only a relatively short timeframe available for the interviews. This limited the ability to derive specific and multifarious information on individual exposures and outcomes. For example, requirements of manual handling operations were only covered by one short question with a limited spectrum of answers. MHO, however, is related to a wide range of different physical demands, including heavy physical work and precise manual work. In the survey, different requirements (high dexterity; applying great strength with the hands and arms; repetitive activities) of manual work are covered by one question. To understand which activity has the strongest influence on the hands and forearms, the workload assessment needs to be based on objective workplace risk assessments and measurements. Obtaining sufficient information on work-related exposure is one of the most challenging quality problems epidemiological studies face. In their review, da Costa et al. [3] discussed how studies that investigate the risk factors for the development of work-related musculoskeletal disorders should report exposure levels in detail. They added that if repetition is identified as a risk factor, the number of repetitions necessary for it to become a risk should be reported as accurately as possible. The expenditure required to obtain information on exposure in such detail is immense. Even in ergonomic field studies, which perform deep ergonomic workplace evaluations with regard to MHO [34, 35], exposures are only assessed at a workplace level, not for individual employees, and not retrospectively. Since such deep exposure assessments are not applicable in large telephone surveys such as the *2018 BIBB/BAuA Employment Survey*, we have to consider the limitations of information related to self-assessments and single-item measurements of exposure and confounding variables. A more advanced approach asking the major key indicators characterizing manual handling operations (duration per day, repetitions, force requirements, or arm posture) should be used to face the problem of mono-methods-bias. It should be noted that the manual handling operations are linked not only with short-term effects like discomfort, but also with specific diseases as long-term outcomes. Here, dose–response relationship between cumulative occupational exposure to manual handling operations, like force and repetition and CTS could be shown [36].

It is also necessary to discuss the wording of the questions on work-related exposures and health outcomes in *BIBB/BAuA Employment Surveys*. Employees were only able to choose between the categories “never”, “rarely”, “sometimes” and “often” to rate the intensity of manual handling operations and other work-related aspects. The categories employed here cannot be linked to an actual time of exposure or an absolute frequency per day. Employment surveys conducted in other countries, such as Denmark, Norway, and Spain, on the other hand, obtain information on parts of the working day [37]. This also addresses the issue that most of the items used in the *BIBB/BAuA Employment Surveys* were proprietarily developed.

However, surveys can assess the outcome in a more differentiated manner. When asked whether they had experienced pain in the hands within the last 12 months, participants only had the option of answering with “yes” or “no”. It is not possible to rate the intensity or frequency of reported pain within the last 12 months in this manner. We can also assume that the ability to remember pain episodes and intensities varies [38]. On the other hand, the questions on health outcomes should be improved. The anatomical regions of “hands” and “arms” are not sufficiently specific to obtain precise information on complaints in hands, forearms, elbows, and shoulders. The general wording of the question is unfortunately conditional to the occurrence of pain while at or after work. At this point, we suggest a more specific and unconditional operationalization of health outcomes in *BIBB/BAuA Employment Surveys*.

Furthermore, a limitation of the study is that important confounders were not assessed and could not be considered in our analysis. On the one hand important occupational factors for hand pain, like hand-arm vibrations and on the other hand important individual factors like body height and weight, and smoking behavior were missing. Future *BIBB/BAuA Employment Surveys* should consider these aspects.

Poisson regression with robust variance estimation was chosen to directly estimate the prevalence ratio as a directly interpretable effect estimate, although the outcome variable is binary and would implicate a logistic regression approach. According to Barros et al. [39], analyses that use Poisson regression with robust variance estimation produce the same results as analyses that use binomial logistic regression. Chen et al. [40] also showed that a robust variance estimation can handle outliers. This allows us to consider a relatively large number of confounders within the models.

Due to the low number of missing data, we chose a complete-case analysis and assumed that missing values occurred completely at random [31].

The sixth *European Working Conditions Survey*, conducted by Eurofound in 2017 [5], and the *2012 BIBB/BAuA Employment Survey* report a similar prevalence of MHO. Moreover, the results of the study support the idea that the German working population is still frequently exposed to MHO. Furthermore, the prevalence of pain in the hands deviates just slightly from the results of the earlier *BIBB/BAuA Employment Survey* in 2012. We should consider, that new and evaluated tools are available to assess workplace risks in the case of manual handling operations [34]. Additionally, Germany implemented a binding offer for preventive occupational health consultations for employees exposed to manual handling operations and other physical workloads in 2013 [41]. Beside this, the Joint German Occupational Safety and Health Strategy (GDA, www.gda-portal.de) put into practice specific workplace prevention programs to reduce the negative impact of physical workload at national level since 2008. The findings additionally coincide with the results of Balogh et al. [18], namely that there is a higher prevalence of disorders in the hand among women. The different ways of assessing MHO make it difficult to compare the results of this study to others. The estimated high positive association between MHO and pain in the hands for the adjusted model are in accordance with the findings of the systematic reviews of Palmer et al. [1] and van Rijn et al. [2].

## Conclusion

In conclusion, the results presented here show that manual handling operations are still a significant occupational exposure in the German workforce. The *2018 BIBB/BAuA Employment Survey* confirmed the known association between MHO and pain in the hands in current working conditions. Employees who “often” perform manual handling operations are especially affected. Therefore, the necessity of prevention measures for occupational groups that often execute MHO is high. These groups include employees in the agricultural sector, services as well as unskilled and skilled manual occupations. The results of this study can be used to justify needs for prevention, to focus on highly affected occupations and to optimize preventive approaches. By setting policies and implementing them within companies, we can reduce the negative impact of MHO on the health of employees. Although according to the reviews of van Eerd et al. [42] and Verhagen et al. [43] evidence about the effectiveness of interventions in the prevention of upper extremity musculoskeletal disorders and symptoms is limited, the implementation of automated or semi-automated processes may reduce the physical workload [44]. In light of the attributable fraction among the exposed, the results suggest a reduction target of at least ten percentage points for pain in the arms and hands if the (self-reported) frequency of manual handling operations were to be reduced from “often” to “never”.

## Supplementary Information

**Additional file 1: Additional Table 1.** Number of missing values per item after applying of selection criteria considering subjects aged <67 and at least 35 h weekly working time.

## Data Availability

The complete dataset of *the 2018 BIBB/BAuA Employment Survey* supporting the conclusions of this article is available as open Scientific-Use-File (no. ZA7574) and can be requested at “BIBB – Bundesinstitut für Berufsbildung—Forschungsdatenzentrum” (PO box 201,264; D-53142 Bonn; Germany; fax number: + 49–(0)228–107–2020); https://www.bibb.de/de/120401.php). The dataset will be available as ftp-download after approved application.

## Abbreviations

BAuA: Federal Institute for Occupational Safety and Health
BIBB: Federal Institute for Vocational Education and Training
CI: Confidence interval
EU-OSHA: European Agency for Safety and Health at Work
MHO: Manual handling operations
MSD: Musculoskeletal disorders
PR: Prevalence ratio
SD: Standard deviation
