# Supplementary material for: The association between manual handling operations and pain in the hands and arms in the context of the 2018 BIBB/BAuA Employment Survey
Source: BMC Musculoskelet Disord. 2021 Jul 30;22:644. doi: 10.1186/s12891-021-04495-z (PMC8323324; doi:10.1186/s12891-021-04495-z)
Supplement: Supplementary file 1 — Additional file 1: Additional Table 1. Number of missing values per item after applying of selection criteria considering subjects aged <67 and at least 35 h weekly working time. [file 12891_2021_4495_MOESM1_ESM.docx]

Additional material

Additional Table 1: Number of missing values per item after applying of selection criteria considering subjects aged <67 and at least 35 hours weekly working time.

| Variable considered in the complete case analysis | Number of subjects available | Missing values per item | Remaining subjects with valid items |
| --- | --- | --- | --- |
| Items used as selection criteria: |  |  |  |
| - Age (<67 years) | 14,414 | 0 | 14,414 |
| - Weekly working hours (>= 35h) | 14,414 | 0 | 14,414 |
| Other items: |  |  |  |
| - Gender | 14,414 | 0 | 14,414 |
| - Psychosocial workload index | 14,414 | 25 | 14,389 |
| - Prevalence of hand pain | 14,414 | 29 | 14,385 |
| - Prevalence of arm pain | 14,414 | 27 | 14,387 |
| - Manual handling operations | 14,414 | 16 | 14,398 |
| - Manual lifting of heavy loads | 14,414 | 6 | 14,408 |
| - Overhead operations | 14,414 | 10 | 14,404 |
| - Cold; heat; wet humidity; draught | 14,414 | 14 | 14,400 |
| Total | 14,414 cases  available after application of the selection criteria | 105 cases  with missing values in any variable | 14,299 complete cases  used in the complete case analysis |
